# Novel pathways of transcriptional and post-transcriptional regulation of post-integrative HIV-1 latency

**DOI:** 10.1186/1758-2652-13-S3-O4

**Published:** 2010-11-04

**Authors:** A Marcello, A Kula, M Dieudonne, A Knezevich, P Maiuri

**Affiliations:** 1 International Centre for Genetic Engineering and Biotechnology, Trieste, Italy

## Background

Therapeutic targeting of HIV latency requires an understanding of the basic mechanisms regulating viral quiescence and activation.

## Methods

We identified several levels of transcriptional and post-transcriptional control of viral genes expression. By looking at the organization of chromatin at the site of integration by chromatin conformation capture and *in situ* hybridization, we found that chromatin imposes silencing in a highly specific spatial and temporal pattern. We also identified several cellular factors that interact with viral RNA by a proteomic approach, pointing to post-transcriptional processes that have been greatly overlooked as mechanisms of HIV post-integrative latency.

## Results

HIV-1 can integrate within active genes at the periphery of the nucleus, and silencing may involve repression from a peri-centromeric region in trans. Hence, a mechanism of active spatial reorganization of chromatin at the site of proviral integration may be ultimately responsible for virus silencing. Particularly relevant to flushing therapies of the viral reservoir is the observation that the silent state of an integrated provirus in a clonal population of activated lymphocytes is not homogeneous, with a small number of cells carrying a provirus embedded into heterochromatin, compared with the majority of cells where the provirus is already poised for transcription.

Proteomic analysis revealed several novel cellular factors, including PSF, MATR3 and p54^nrb^ that have been previously in nuclear retention of RNA. We demonstrated that PSF/p54^nrb^ binds nascent HIV RNA at the transcription site, but MATR3 defines a novel sub-nuclear compartment where this viral RNA is retained. Rev is able to associate with unspliced HIV RNA co-transcriptionally directing its nuclear export. These observations lead to a model for a novel cellular pathway of RNA retention that is hijacked by the virus.

## Conclusions

Both the control imposed by the organization of the nucleus and by post-transcriptional mechanisms is relevant for the control of HIV-1 post-integrative latency.

